# Anthocyanins from *Lycium ruthenicum* Murray attenuates high‐fat diet‐induced hypercholesterolemia in ApoE ^−/−^ mice are related to the modulation of gut microbiota and the ratio of conjugated to unconjugated bile acids in fecal bile acid profile

**DOI:** 10.1002/fsn3.3923

**Published:** 2023-12-26

**Authors:** Meng Zhang, Hui Li, Tingting Tan, Lu Lu, Jia Mi, Abdul Rehman, Yamei Yan, Linwu Ran

**Affiliations:** ^1^ Key Laboratory of Environmental Factors and Chronic Disease Control, School of Public Health Ningxia Medical University Yinchuan China; ^2^ Goji berry Research Institute Ningxia Academy of Agriculture and Forestry Sciences Yinchuan China; ^3^ School of Clinical Medicine Ningxia Medical University Yinchuan China

**Keywords:** bile acids, gut microbiota, hypercholesterolemia, *Lycium ruthenicum* Murray

## Abstract

Previous findings showed that anthocyanins from *Lycium ruthenicum* Murray (ACN) reduced HFD‐induced hypercholesterolemia by regulating gut microbiota, but the mechanism has not been fully understood. The objective of this research was to know whether the cholesterol‐lowering impact of ACN in HFD‐induced ApoE^−/−^ mice is related to the gut microbiota‐bile acid (BA) metabolism. Twenty‐four male ApoE^−/−^ mice were divided into three groups: the Control group, the HFD group, and the HFD + ACN group. Here, we showed that ACN intervention reduced HFD‐induced body weight serum concentrations of TC and LDL‐C and ameliorated lipid accumulation in the liver and adipose tissues. Besides, ACN altered gut microbiota composition in HFD‐fed ApoE^−/−^ mice. Moreover, UHPLC–MS/MS analysis revealed that ACN intervention significantly increased the ratio of conjugated to unconjugated BAs in feces induced by HFD, attributed to the increase in conjugated BAs and decrease in unconjugated BAs. Finally, the correlation analysis indicated that the above changes in fecal BA profile were linked with an increase in *Bifidobacterium*, *Allobaculum* and a decrease in *Ileibacterium*, *Helicobacter*, *Rikenellaceae_RC9_gut_group*, *Blautia*, *Odoribacter*, and *Colidextribacter*. In summary, ACN could alleviate HFD‐induced hypercholesterolemia in ApoE^−/−^ mice, which was associated with the improvement of gut microbiota and modulation of fecal BA profile.

## Graphical abstract

Anthocyanins from *Lycium ruthenicum* Murray (ACN) could alleviate hypercholesterolemia in high‐fat diet‐induced ApoE^−/−^ mice by improving the gut microbiota and modulating fecal bile acid (BA) profile, especially the ratio of conjugated to unconjugated BAs. This research provides a new theoretical basis for ACN to alleviate metabolic diseases and cardiovascular diseases.

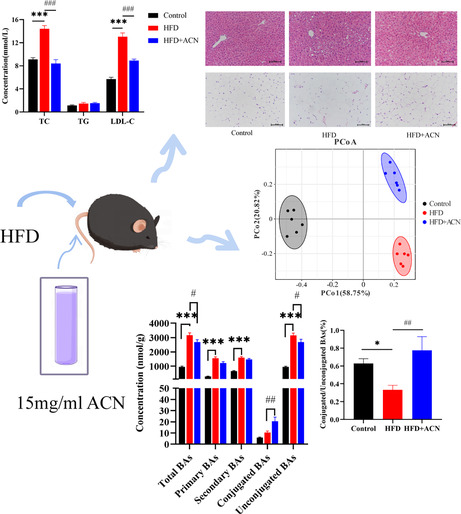

## 1 INTRODUCTION

Atherosclerosis (AS), an inflammatory disease affecting large arteries, remains a significant factor in the occurrence of cardiovascular disease (CVD) globally (Björkegren & Lusis, 2022). Hypercholesterolemia often acts as an early indicator of atherosclerosis (Zhu et al., 2013). Avoiding the danger of CVD requires the decrease in total cholesterol (TC) and low‐density lipoprotein cholesterol (LDL‐C) levels in bloodstream during hypercholesterolemia (Vourakis et al., 2021). Numerous experimental and epidemiological research have proven that taking foods abundant in polyphenols and dietary supplements can effectively decrease the risk of developing CVD (Scalbert et al., 2005). Clinical trials and animal studies suggested that polyphenols could modify glucose and lipid metabolism (Matacchione et al., 2020; Ramos et al., 2023; Wang, Ma, et al., 2022). Berries are well‐described as interesting sources of polyphenols, especially anthocyanins (de Souza et al., 2014).

According to the reports, anthocyanins demonstrate positive effects in counteracting the impact of unhealthy diets by regulating dysmetabolism and modifying glycemia and lipidemia (Cremonini et al., 2022; Sivamaruthi et al., 2020). *Lycium ruthenicum* Murray, an emerging economic plant resource in northwestern China, has been recognized for its diverse biological functions attributed to its rich anthocyanin content (Tang et al., 2017). In recent studies, anthocyanins from *Lycium ruthenicum* Murray (ACN) contribute to weight reduction, influence lipid metabolism, modulate gut microbiota, and have antioxidant and anti‐inflammatory effects (Peng et al., 2019; Xie et al., 2018). The bioactivity of anthocyanins is believed to be linked to the gut microbiota, given the notably low bioavailability of anthocyanins (Santhakumar et al., 2018). Recent research revealed that prolonged consumption of ACN promoted the entire organism health by enhancing antioxidant and anti‐inflammatory compounds, maintaining the intestines, and positively influencing gut microbiota (Peng et al., 2020). And Li, Liu, et al. (2022) confirmed that ACN has the potential to effectively modulate gut microbiota to ameliorate high‐fat diet (HFD) ‐induced dyslipidemia in C57/BL6J mice. However, the specific mechanisms by which ACN achieves its lipid‐lowering effects through regulation of the gut microbiota remain unclear.

The gut microbiome positively affects the host's physiological procedure by generating a diverse array of metabolites. These metabolites perform as signaling molecules and serve as substrates for numerous metabolic reactions that occur within the host (Krautkramer et al., 2021). Research involving both humans and mice has shown that obesity and related diseases change the configuration of gut microbiota, which, in turn, provide the phenotypes of these diseases (Arora & Bäckhed, 2016). Metabolites generated by the gut microbiota hold a key importance in regulating metabolism in the host (Yang & Cong, 2021). Bile acids (BAs) represent the primary downstream metabolites of cholesterol (Xiao et al., 2018). Hence, regulating the BA profile is essential for cholesterol homeostasis. Within the intestine, gut microbes actively alter BA structure and function (Jia et al., 2018). The deconjugation of BA is catalyzed by bacteria that possess bile salt hydrolase (BSH) activity (Wahlström et al., 2016). As reported, some biologically active substances could ameliorate lipid metabolism by inhibiting the activity of BSH (Huang et al., 2019; Zhong et al., 2023). The interaction between the gut microbiota and BAs is of crucial significance in the control of energy extraction, lipid processing, and maintenance of cholesterol and BA equilibrium (Sayin et al., 2013).

Apolipoprotein E (ApoE) is a carrier protein for lipids. It plays a crucial role as a ligand for LDL receptors, thereby greatly contributing to cholesterol metabolism and the progression of AS. The presence of ApoE mutation or deficiency has been shown in multiple population‐based studies to exacerbate dyslipidemia and hasten the progression of AS (Ghiselli et al., 1981). The effect of ACN intervention on improving the gut microbiota composition of ApoE^−/−^ mice and the mechanisms involved have not been reported yet. Building upon our prior research, we delved into whether the cholesterol‐reducing impact of ACN in HFD‐induced ApoE^−/−^ mice was involved in the alteration of gut microbiota and BAs or their relationships. 16S rRNA sequencing was utilized to determine the gut microbiota structure and UHPLC–MS/MS analysis was utilized to quantify and characterize fecal BAs. We then used correlation analysis to identify a connection between gut microbiota and fecal BAs, offering novel insights into potential mechanisms that underlie the preventive effects of ACN on hypercholesterolemia.

## 2 MATERIALS AND METHODS

### 2.1 Materials and reagents

The high‐fat diet MD12015 was bought from the Medicine Company in Yangzhou, China. The control diet was bought from Xietong Pharmaceutical Bio‐engineering company in Nanjing city of China. We purchased LDL‐C, TC, and TG kits from Nanjing Jiancheng Bioengineering Institute. The Hematoxylin and eosin (H&E) solution for staining was procured from Leagene Biotechnology Co., Ltd. in Beijing, China. Reference standards of 33 BAs and six stable isotope‐labeled standards were obtained from ZZ Standards Co., Ltd situated in Shanghai, China. Precisely taurohyodeoxycholic acid (THDCA), taurohyocholic acid (THCA), 7‐ketolithocholic acid (7_ketoLCA), nor cholic acid (NorCA), taurodeoxycholic acid (TDCA), hyodeoxycholic acid (HDCA), glycohyocholic acid (GHCA), 23‐nordeoxycholic acid (23norDCA), isolithocholic acid (isoLCA), 12‐ketolithocholic acid, (12_ketoLCA), allolithocholic acid (alloLCA), lithocholic acid 3‐sulfate (LCA_S), deoxycholic acid (DCA), glycolithocholic acid (GLCA), taurochenodeoxycholic acid (TCDCA), glycoursodeoxycholic acid (GUDCA), chenodeoxycholic acid (CDCA), cholic acid (CA), tauroursodeoxycholic acid (TUDCA), chenodeoxycholic acid‐3‐β‐D‐glucuronide (CDCA_3Gln), lithocholic acid (LCA), 3β‐Ursodeoxycholic acid (βUDCA), glycochenodeoxycholic acid (GCDCA), glycocholic acid (GCA), taurolithocholic acid (TLCA), ursodeoxycholic acid (UDCA), 3‐dehydrocholic acid (3_DHCA), hyocholic acid (HCA), α‐Muricholic acid (αMCA), β‐Muricholic acid (βMCA), taurocholic acid (TCA), tauro‐α‐Muricholic acid (TαMCA), glycodeoxycholic acid (GDCA). Ammonium acetate was of analytical grade and acquired from Sigma‐Aldrich. Optima LC/MS grade methanol, acetonitrile, and formic acid were bought from Thermo‐Fisher Scientific.

### 2.2 Preparation of anthocyanins from the fruits of *Lycium ruthenicum* Murray

The fresh fruits of plant *Lycium ruthenicum* Murray were sourced from the National Wolfberry Engineering Research Center (Yinchuan, China). The separation of ACN was taken according to the reported procedure (Li, Liu, et al., 2022). Concisely, the fruits of *Lycium ruthenicum* were pulverized into a powder, followed by treatment with a methanol solution with 0.5% trifluoroacetic acid (TFA) for half an hour. The mixture obtained as a result underwent a process of vacuum filtration, wherein the extract was gathered and condensed by reducing the pressure at a temperature of 38°C. Subsequently, the condensed solution was subjected to lyophilization to generate the crude ACN extract. The crude extract then underwent an additional purification process, where it was consecutively transferred through a column containing XAD‐7 macroporous resin. Then, the resulting product was concentrated and freeze‐dried to get the final purified ACN. The concentrations of total phenols and anthocyanins in the purified ACN extracts were 26 ± 2.3% and 15.1 ± 1.2%, respectively, using the Folin‐phenol reagent and pH differential method. The identification of the main compounds has been reported in previous research (Li et al., 2021; Li, Liu, et al., 2022; Tang et al., 2017).

### 2.3 Animals studies

We sourced 6‐week‐old ApoE^−/−^ mice from the Lab Animal Center of Ningxia Medical University (Yinchuan, China). These mice were carefully held in a specific pathogen‐free facility that ensured their health and well‐being. The facility maintained a light–dark cycle for 12 h, a temperature of 22 ± 2°C, and an abundant supply of chow and water. After a period of 7 days (1 week), mice were adjusted to new surroundings; they were casually allocated in three groups (*n* = 8). The first was a control group in which they were receiving normal chow and pure water. The second group (HFD) with a high‐fat diet and pure water. Lastly, a third group, HFD with ACN group (HFD + ACN) that receiving HFD and 15 mg/mL ACN solution. Throughout the 16‐week experimental period, mice were provided with no restriction to access food and water, with weekly measurements of body weight. Conclusion of experimental trials: mice were euthanized by a carbon dioxide animal asphyxiator after food restriction for one night. In order to collect blood samples, the inner canthus vein was punctured using 1 mL syringes. These samples then underwent a clotting process at room temperature for half an hour, ensuring that clot was thoroughly completed after clotting centrifugation was carried out at 4°C, 3500 × ***g*** for 10 min to get the serum samples, which then preserved at −80°C for subsequent biochemical examinations. Freshly obtained samples of feces were promptly gathered and subsequently stored at −80°C until the examination of the intestinal microbiota and BA could be conducted. Tissues, including liver and adipose, were weighted, and some sections were fixed in a 4% paraformaldehyde for further histological examination. Prior to commencing any procedures involving animals, ethical approval for all the animal procedures was obtained from the Animal Ethics and Welfare Committee of Ningxia Medical University (IACUC‐NYLAC‐2023‐085).

### 2.4 Measurement of serum lipid levels

The concentrations of TC, TG, and LDL‐C in serum were quantified utilizing commercially available kits following the manufacturer's protocols.

### 2.5 Histological assessment

After dissection of mice, liver and adipose tissues were immersed in a 4% paraformaldehyde solution for a duration of 24 h. The tissue was then dehydrated using an automatic tissue dehydrator ASP300; following dehydration, they were undergoing embedding in paraffin, followed by sectioning at a 5 μm thickness. These sections then underwent H&E staining, involving deparaffinization in xylene, graded ethanol washes, staining with hematoxylin for 4 min, rinsing with 1% acidic ethanol differentiation solution, a quick water immersion for 10 min, and retaining by applying eosin for 2 min. Subsequently, sections were washed in water for 5 min and subjected to a gradient of ethanol and xylene. The H&E‐stained divisions were observed by microscope (Nikon).

### 2.6 16S rRNA gene sequencing

Total genome DNA was derived from fecal samples utilizing the cetyl trimethyl ammonium bromide method. DNA quality was evaluated using agarose gel еlеctrophorеsis. Its concentration was quantified. Subsequently, we performed amplification of 16S rRNA gene V3–V4 region from fecal bacteria. The total PCR reaction system, comprising 30 μL, included a high‐fidelity DNA polymerase premix (15 μL), 0.2 μM of 341F and 806R primers, and 10 ng of wеll‐еxtractеd template DNA. The PCR reaction program followed the method of Wu et al. (2021) with an initial denaturation at 98°C for 1 min, following denaturation at 98°C for 10 s. The procedure of annealing began at 50°C and continued for 30 s, followed by a 30‐s continuation of the primers at 72°C for a total of 30 cycles. Eventually, there was a 5‐min final extension at 72°C. Amplified PCR material was collected and distilled utilizing the Qiagеn Gel Extraction Kit (Qiagеn). Subsequent library construction and sequencing were conducted with support from Novogеnе Co., Ltd.

The obtained sequence information underwent splicing and filtering to obtain valid data. Subsequently, Uparsе software (v7.0.1001) was employed for clustering valid data into operational taxonomic units (OTUs) with a 97% identity threshold. Species classification analysis was carried out using the Silva Database. The standardized OTU abundance table served as the foundation for further data analysis. R software (v4.2.2) was used for sequence data analysis of the 16S rRNA gene. Beta diversity analysis, employing principal coordinate analysis (PCoA) explored the structural changes within the fecal gut microbiota. LEfSе analysis, based on the OTU table, was employed to identify species, emphasizing the significant distinction between groups, with the LDA score threshold set to 4.

### 2.7 BAs analysis

BA standard substances were weighed and a linear master mixture was prepared with a concentration of 25 μg/mL. The linear master mixture was then diluted with methanol to generate working solutions at concentrations of 25,000, 15,000, 5000, 2500, 500, 500, 250, 250, 50, 25, 15, 5, 2.5, or 1.5 ng/mL. BAs were analyzed using UHPLC–MS/MS (QTRAP 6500+, AB SCIEX Corp.), with a system set up with a Waters ACQUITY UPLC BEH C18 column (2.1 × 100 mm, 1.7 μm). The method was adapted from Wang, Han, et al. (2022) with certain adjustments. Fecal samples (50 mg) were resuspended and then added to water by vortexing as the 10 times diluted sample. Subsequently, 100 μL of the previously diluted sample was obtained and added to 300 μL of a precipitating solution (acetonitrile: methanol = 8:2) comprising 0.05 μM mixed internal standards. The mixture was then centrifuged at 10,000 × ***g*** for 10 min. We collected the supernatant for further examination. A 2 μL sample was еlutеd through the column at 50°C at a flow rate of 300 μL/min, utilizing conditions for elution as initial 20% acetonitrile (B) and 0.1% formic acid/water (A) for 0.5 min; 20–35% B for 0.5 min; 35–37% B for 1.5 min; 37–38% B for 1.6 min. Subsequently, it was held at 38% B for 0.9 min, then by 38–39% B for 1 min; 39–40% B for 0.5 min, 40–44% B for 2 min; 44–45% B for 0.5 min; 45–52% B for 0.5 min; 52–65% B for 3 min, 65–100% B for 2.5 min, and 100–20% B for 2 min. The entire linear gradient program took 17 min. BAs were analyzed through scheduled multiple reaction monitoring (MRM) with a mass spectrometer. Following the parameters as IonSpray Voltage (−4500 V), Curtain Gas (35 psi), Ion Source Temp (550°C), Ion Source Gas of 1 and 2 (60 psi).

A standard curve was established with standard substance concentration as the *x*‐axis and the ratio of peak area of standard substance to internal standard substance as the *y*‐axis. Subsequently, analysis and calculation of fecal BA components were conducted. Linearity was considered acceptable within the concentration range with a correlation coefficient (*R* ^2^) of ≥.99. And precision, accuracy, and stability were maintained, with a relative standard deviation of ≤15%. After handling missing values, principal component analysis (PCA) was carried out on fecal BAs, helping to describe overall metabolic differences between groups and variations within samples within the same group.

### 2.8 Statistical analysis

Data were noted as the mean ± SEM. Two‐way repeated‐measurement ANOVA was used for data analysis from weekly body weight using SPSS23.0 software. Statistical value between groups was assessed through one‐way ANOVA or Kruskal–Wallis test. The significance *p*‐value less than .05 was evaluated as statistically significant, and the least significant difference (LSD) test was applied when the *p*‐value fell below .05.

## 3 RESULTS

### 3.1 ACN supplementation reduced HFD‐induced body weight in ApoE ^−/−^ mice

**FIGURE 1:**
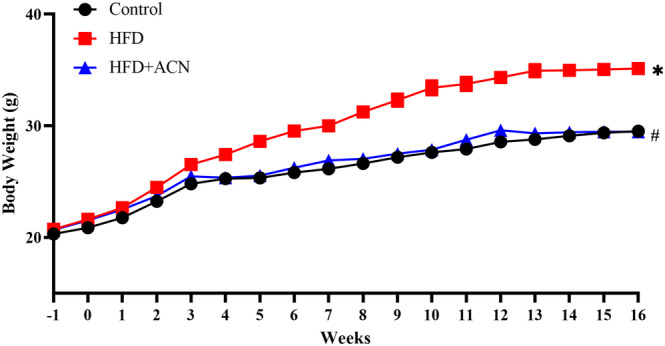
Effects of ACN on body weight in HFD‐fed ApoE^−/−^ mice. ApoE^−/−^ mice were receiving normal chow and pure water (Control group); ApoE^−/−^ mice were receiving HFD without (HFD group) or with 15 mg/mL ACN solution (HFD + ACN group). Values were presented as mean ± SEM. *p < .05 versus Control group; ^#^ *p* < .05 versus HFD group.

Following a 16‐week HFD treatment, the weight of ApoE^−/−^ mice fed with HFD exhibited notable weight gain compared to control mice. After 16 weeks of ACN supplementation through their drinking water, the HFD + ACN group showed significant weight loss compared to ApoE^−/−^ mice fed HFD (Figure 1).

### 3.2 ACN effects on the levels of serum lipid in HFD‐induced ApoE ^−/−^ mice

**FIGURE 2:**
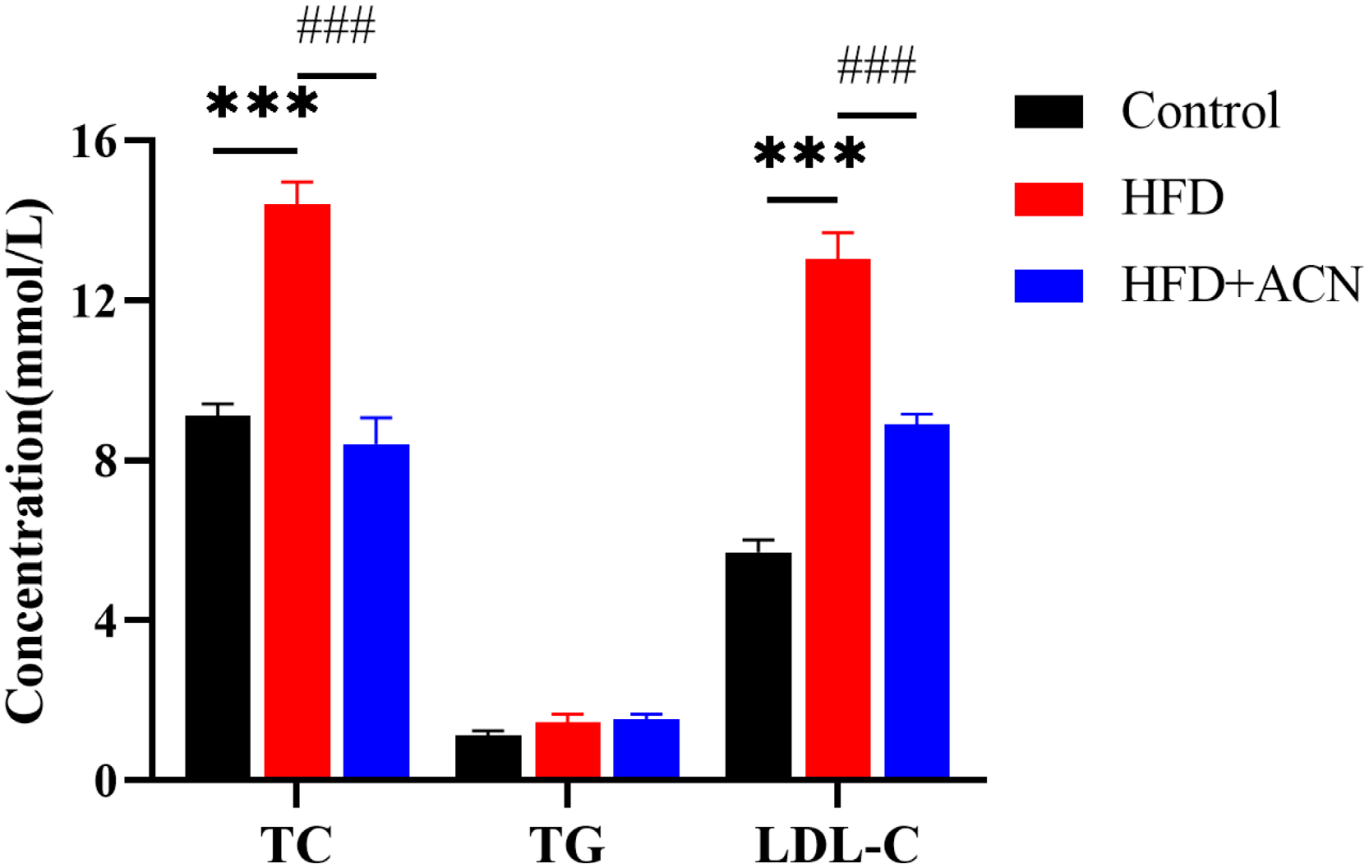
Effects of ACN on the serum lipid levels in HFD‐fed ApoE^−/−^ mice. Values were presented as mean ± SEM. *p < .05 or ***p < .001 versus Control group; ^#^ *p* < .05 or ^###^ *p* < .001 versus HFD group.

An HFD feeding was found to significantly increase the serum levels of TC and LDL‐C. The HFD + ACN group displays notably lower TC and LDL‐C levels compared to the HFD group. There is no statistically significant distinctiveness in TG levels between the three groups (Figure 2). These findings suggested ACN supplementation could ameliorate HFD‐induced dyslipidemia in ApoE^−/−^ mice.

### 3.3 ACN supplementation ameliorated HFD‐induced accumulation of lipids in the liver and adipose tissues of ApoE ^−/−^ mice

**FIGURE 3:**
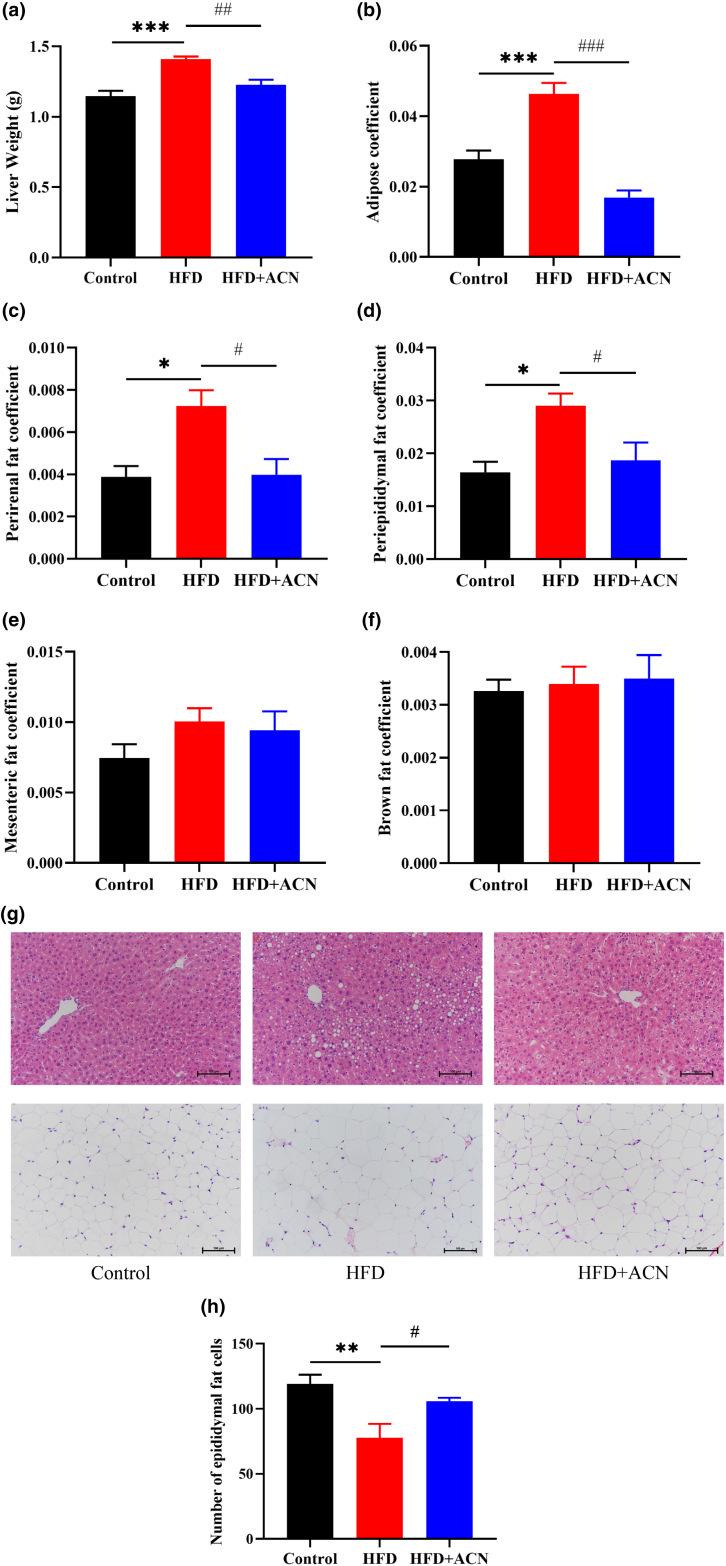
Effects of ACN supplementation on liver and adipose tissues of ApoE^−/−^ mice fed with HFD. (a) weight liver; (b) adipose coefficient; (c) mesenteric fat coefficient; (d) perirenal fat coefficient; (e) brown fat coefficient; (f) peri‐epididymal fat coefficient; (g) H&E staining of liver and peri‐epididymal fat tissues (200×); (h) number of peri‐epididymal fat cells. Values were presented as mean ± SEM. *p < .05 or ***p < .001 versus Control group; ^#^ *p* < .05, ^##^ *p* < .01 or ^###^ *p* < .001 versus HFD group.

The liver and adipose tissues were weighed to ascertain the potential impact of ACN supplementation on body weight reduction in ApoE^−/−^ mice induced by HFD. The aim was to determine whether ACN supplementation led to a reduction in body weight with respect to fat loss. As shown in Figure 3a,b, the increased weight of liver and adipose coefficient in HFD‐induced ApoE^−/−^ mice were considerably enhanced compared to the Control group, but the HFD + ACN group effectively prevented the HFD‐induced increases in liver weight and adipose coefficient in ApoE^−/−^ mice.

We further calculated specific adipose tissue coefficients, including perirenal fat, peri‐epididymal, mesenteric, and bilateral perirenal fat coefficients. Figure 3e,f revealed no considerable distinctiveness within the three groups in the mesenteric fat and brown fat coefficients. However, the perirenal fat coefficient and peri‐epididymal fat coefficient exhibited considerably greater in the HFD group compared to the Control group. Interestingly, the perirenal fat coefficient and peri‐epididymal fat coefficient were revealed to be significantly lower in the HFD + ACN group than in the HFD group (Figure 3c,d).

Histopathological examination through H&E staining depicted clear central veins and hepatic strands with well‐defined boundaries of the hepatic lobules within the liver of the Control group. The liver of the HFD group exhibited evident lipid droplet accumulation, disturbed arrangement of hepatic cords, and blurred boundaries of the hepatic lobules. However, those pathological changes were remarkably alleviated by ACN supplementation. Besides, ACN notably ameliorated HFD‐induced fat accumulation and fat cell expansion in peri‐epididymal fat cells (Figure 3g,h). These data suggested that ACN supplementation could ameliorate fat deposition and body weight of ApoE^−/−^ mice induced by HFD.

### 3.4 ACN supplementation altered the composition of gut microbiota in HFD‐fed ApoE ^−/−^ mice

**FIGURE 4:**
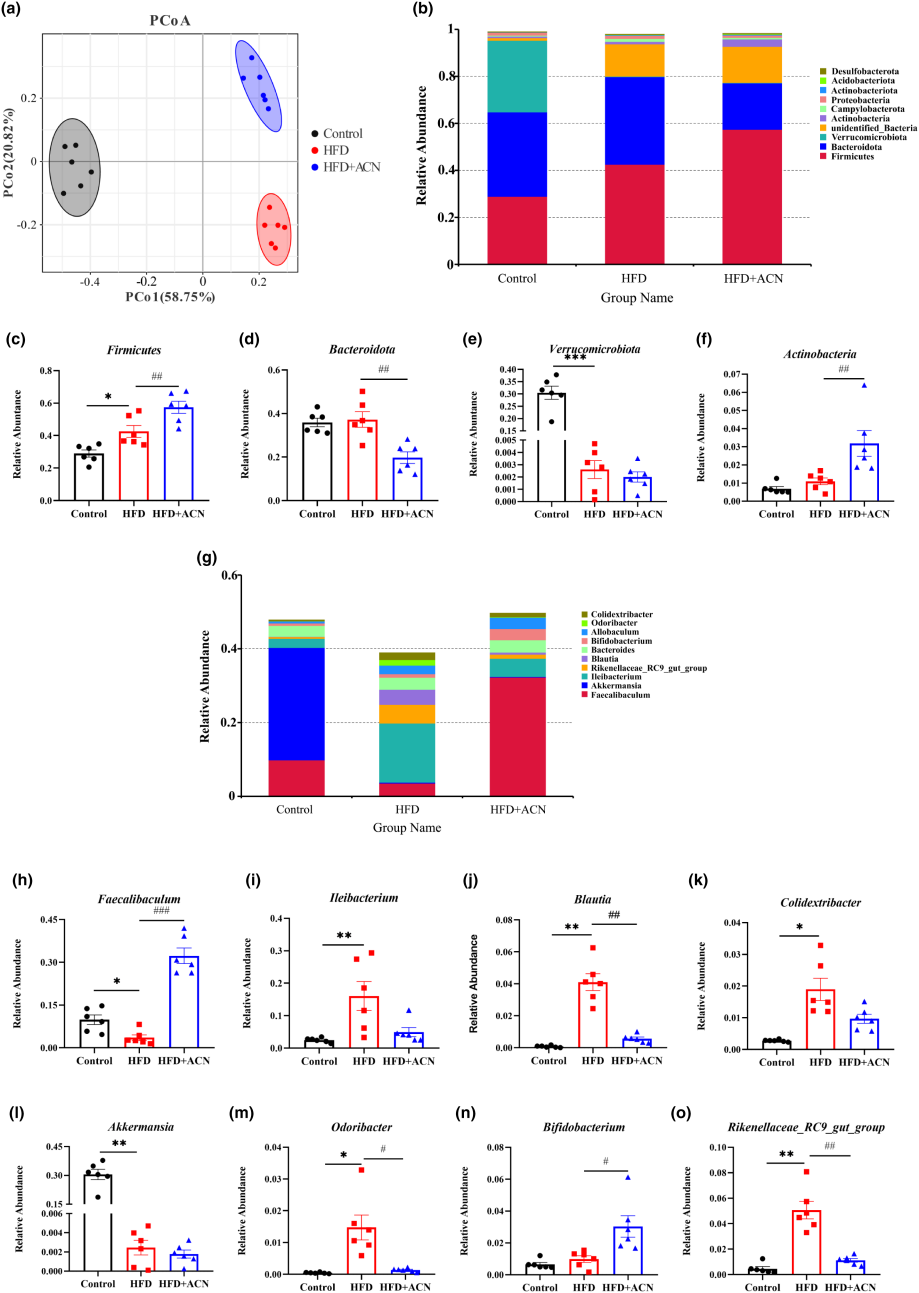
ACN altered the composition of gut microbiota in HFD‐fed ApoE^−/−^ mice. (a) PCoA based on OTUs level; (b) microbial community composition at phylum level; (c–f) relative abundance of major bacteria at phylum level; (g) microbial community composition at phylum level; (h–o) relative abundance of differential bacteria at genus level. Data were presented as mean ± SEM. *p < .05 or **p < .01 versus Control group; ^#^ *p* < .05, ^##^ *p* < .01 versus HFD group.

Overall alterations in gut microbiota structure due to ACN supplementation were assessed by analysis of 16S rRNA gene sequences of microbial samples taken from feces. PCoA analysis based on OTUs demonstrated a considerable response in gut microbial composition to both HFD and ACN interference (Figure 4a). The gut microbial configuration in the HFD group showed complete segregation from the Control group. As expected, the HFD + ACN group was clearly distinguished from the HFD group, suggesting that ACN supplementation could ameliorate the intestinal flora disturbances induced by HFD in ApoE^−/−^ mice.

The bacterial abundance at various taxonomic levels was compared across groups to delve deeper into the specific alternation in bacterial communities. As shown in Figure 4b–f four dominant phyla (*Firmicutes*, *Bacteroidota*, *Verrucomicrobiota*, and *Actinobacteria*) collaborate among all groups with varying relative abundance. Compared to the Control group, the relative abundance of *Firmicutes* noticeably increased, and the relative abundance of *Verrucomicrobiota* noticeably decreased in the HFD group. Meanwhile, relative abundances of *Firmicutes* and *Actinobacteria* noticeably increased, and relative abundance of *Bacteroidota* noticeably decreased were also noted in the HFD + ACN group in comparison to the HFD group. On genus level, the HFD group displayed higher relative abundances of *Ileibacterium*, *Blautia*, *Colidextribacter*, *Rikenellaceae_RC9_gut_group*, and *Odoribacter* and relative abundances reduction of *Faecalibaculum*, *Akkermansia*, comparison to the Control group (Figure 4g–o). However, compared to the HFD group, ACN supplementation reversed relative abundances of bacteria mentioned above, such as *Faecalibaculum*, *Blautia*, and *Rikenellaceae_RC9_gut_group*. In addition, ACN supplementation also significantly enhanced the relative abundance of *Bifidobacterium*. The gut microbial composition in our findings showed that ACN significantly ameliorated the gut microbiota disturbances caused by HFD in ApoE^−/−^ mice.

**FIGURE 5:**
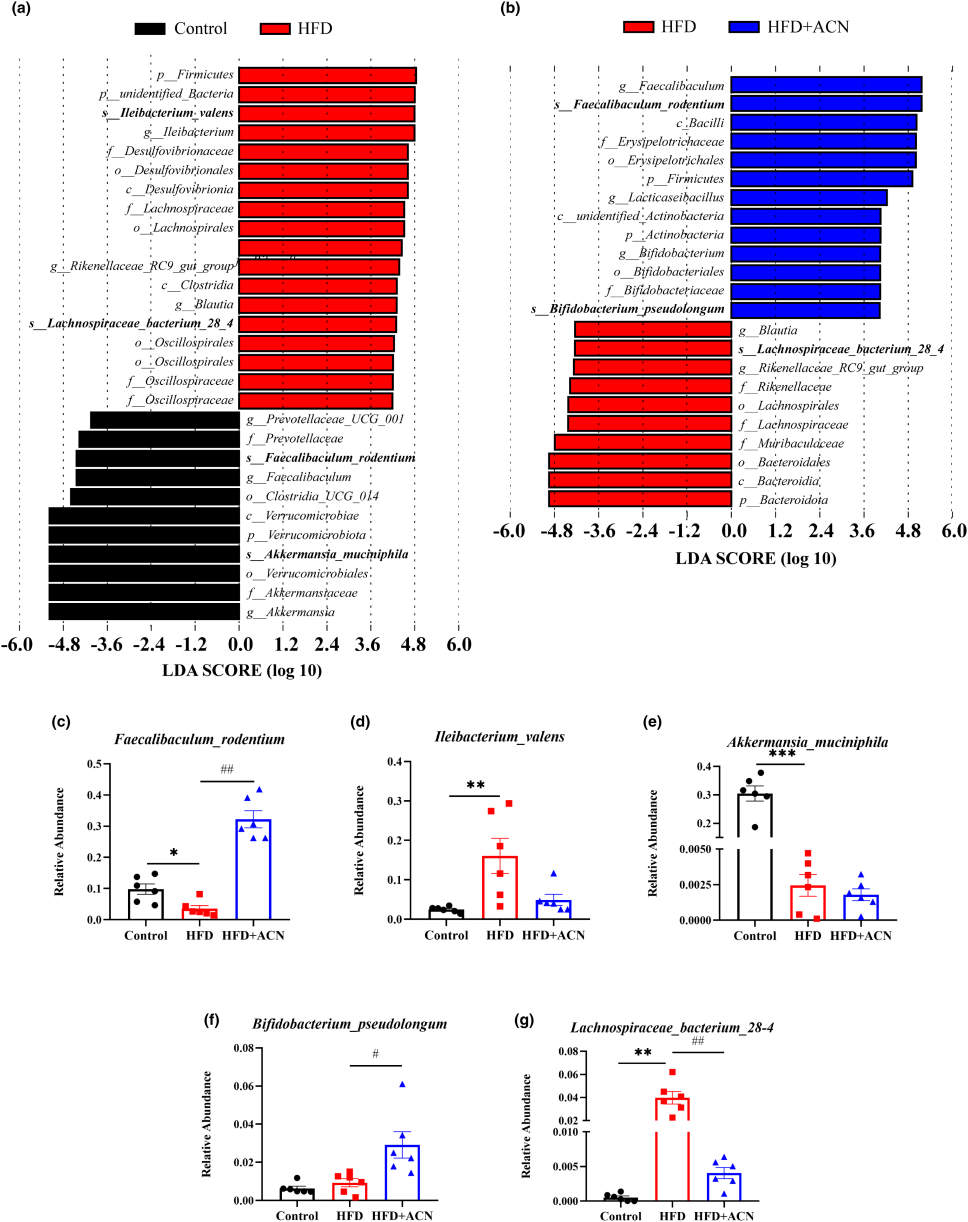
(a, b) gut microbiota comparisons between groups analyzed by LEfSe at different taxonomy levels, with taxa meeting LDA score threshold >4 being listed; (b–f) Relative abundance of major bacteria at species level. Data were presented as mean ± SEM. *p < .05, **p < .01 or ***p < .001 versus Control group; ^#^ *p* < .05, ^##^ *p* < .01 versus HFD group.

LEfSe analysis was utilized for the identification of specific gut microbial taxa. Compared to the HFD group, the Control group displays dominance of *Akkermansia_muciniphila* and *Faecalibaculum_rodentium* at species level. On the contrary compared to the Control group, the HFD group showed a dominant presence of *Ileibacterium‐valens* and *Lachnospiraceae_bacterium_28–4* at species level. Compared to the HFD group, the HFD + ACN group featured the species *Faecalibaculum_rodentium* and *Bifidobacterium_pseudolongum* (Figure 5). These results were consistent with characteristics at the genus level.

### 3.5 ACN supplementation altered the fecal BA profile in HFD‐fed ApoE ^−/−^ mice

**FIGURE 6:**
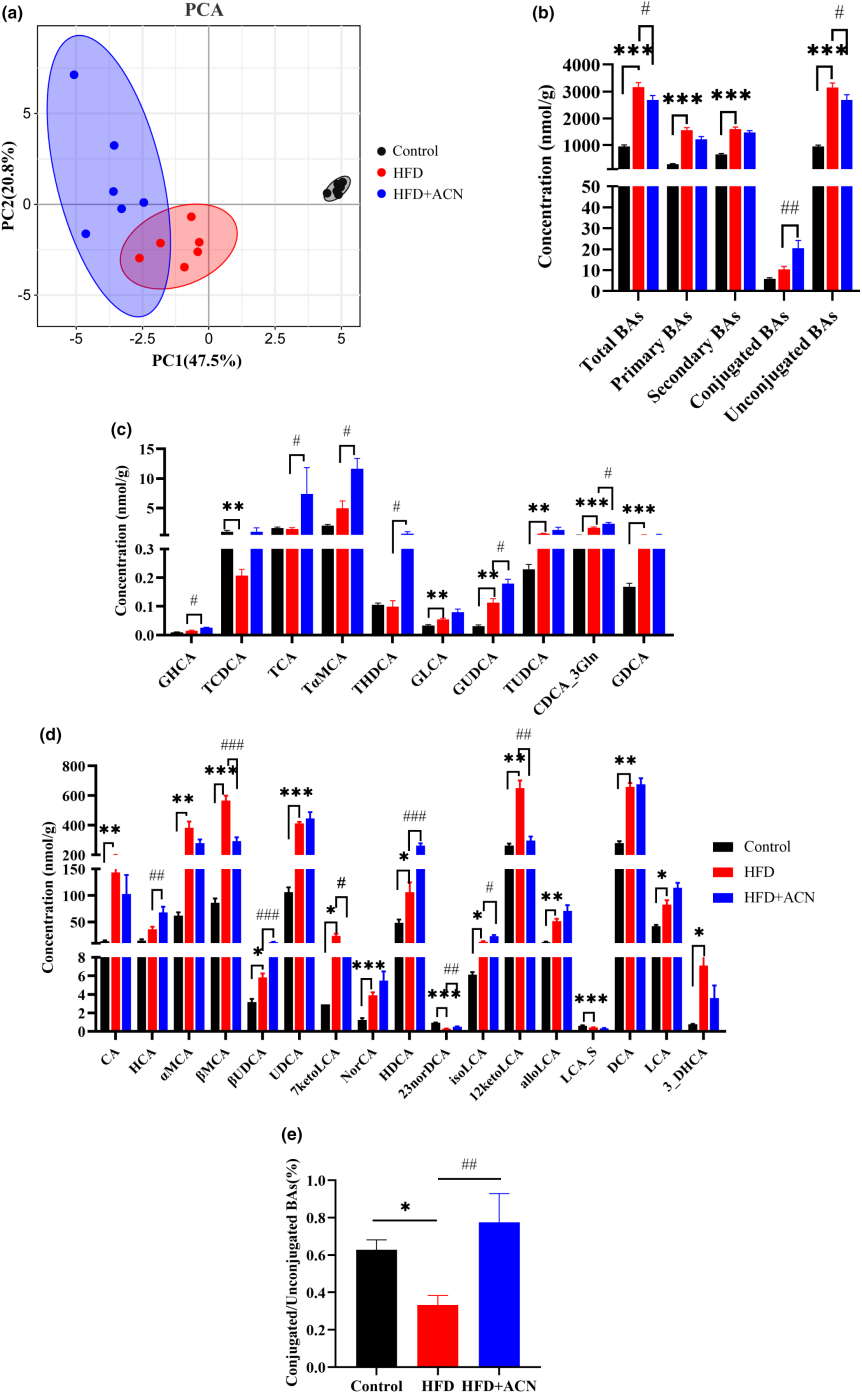
ACN altered the BA profiles in HFD‐fed ApoE^−/−^ mice. (a) principal component analysis (PCA) of the feces BAs; (b) fecal BA classes; (c) concentrations of conjugated BAs in feces; (d) concentrations of conjugated BAs in feces; (e) ratio of conjugated BAs to unconjugated BAs in feces. Data were presented as mean ± SEM. *p < .05, **p < .01 or ***p < .001 versus Control group; ^#^ *p* < .05, ^##^ *p* < .01, or ^###^ *p* < .001 versus HFD group.

We employed a target metabolomics approach using UHPLC–MS/MS to analyze BAs in the feces of the ApoE^−/−^ mice. The PCA plot clearly illustrated distinct fecal BA composition clustering among the experimental groups (Figure 6a). Figure 6b demonstrated the conclusions that the total BAs concentration, primary BAs level, and secondary BAs level in feces were considerably increased in the HFD group compared to the Control group. Furthermore, levels of unconjugated BAs, such as CA, αMCA, βMCA, UDCA, 12keto_LCA, and DCA, were also increased in the HFD group compared to the Control group. Anyhow, the unconjugated BAs, including βMCA and 12keto_LCA, were significantly decreased, and conjugated BAs levels, including TCA, TαMCA, and THDCA, were increased in the HFD + ACN group in comparison to HFD group (Figure 6c,d). In Figure 6e, the ratio of conjugated to unconjugated BAs reduced considerably in the HFD group compared to the Control group. However, in comparison to the HFD group, a substantial ratio increases from conjugated to unconjugated BAs in the HFD + ACN group.

### 3.6 Correlation between gut microbiota and fecal BA profile in ApoE ^−/−^ mice

**FIGURE 7:**
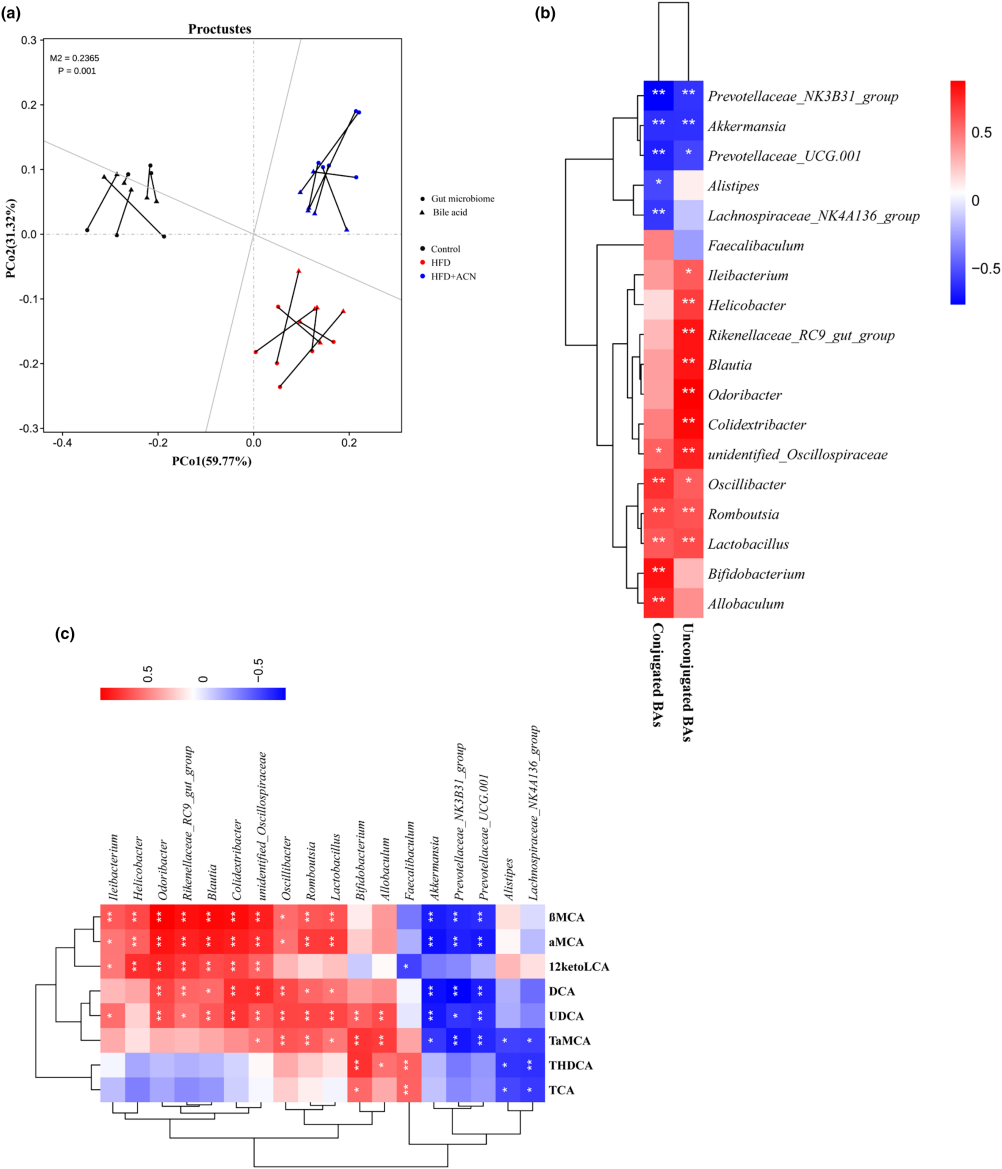
Correlation between gut microbiota and fecal BA profile in HFD‐fed ApoE^−/−^ mice. (a) Procrustes analysis of the gut microbiota and fecal BA data; (b, c) heatmap of spearman correlation coefficients between the gut microbiota and fecal BAs. The gradient colors represent the correlation coefficients, with red color being more positive and blue color indicating more negative. *p < .05; **p < .01.

We further analyzed the correlation between gut microbiota and fecal bile acids. PCoA of gut microbiota and fecal BA data, followed by Procrustes analysis between sample points, showed a significantly high incorporation among gut microbiota and BA in feces between different groups (*M* ^2^ = 0.2365, *p* = .001; Figure 7a). We observed 20 genus‐level correlations with conjugated BAs and unconjugated BAs in feces. As demonstrated in Figure 7b, genera such as *Ileibacterium*, *Helicobacter*, *Rikenellaceae_RC9_gut_group*, *Blautia*, *Odoribacter*, and *Colidextribacter*, which exhibited higher abundance in the HFD group in comparison to the Control or HFD + ACN groups, had positive correlations with unconjugated BAs. The relative abundances of *Allobaculum* and *Bifidobacterium*, which were more abundant in the HFD + ACN group compared to the HFD or Control groups, showed positive correlations with conjugated BAs. According to the differential in BAs analysis among groups, we then performed a correlation analysis using eight high concentrations of differential BAs with gut microbiota, which largely supported a relationship between 20 gut microbiota at the level of genus and conjugated, unconjugated concentrations of BAs (Figure 7c). These findings indicate ACN could regulate the ratio of conjugated to unconjugated BAs in HFD‐induced ApoE^−/−^ mice, was incorporated with decrease in relative abundances of *Ileibacterium*, *Helicobacter*, *Rikenellaceae_RC9_gut_group*, *Blautia*, *Odoribacter*, and *Colidextribacter* and rise of relative abundances of *Allobaculum* and *Bifidobacterium*.

## 4 DISCUSSION

Serum cholesterol levels were prominently crucial in CVD development (Jousilahti et al., 1999), therefore, reducing serum cholesterol levels is essential in preventing and curing CVD. As phytochemicals widely found in plants, anthocyanins have been reported to improve hypercholesterolemia by improving the serum lipid profile, reducing inflammation, and inhibiting fat accumulation (Zhu et al., 2011). In addition, studies have found that ACN and its monomer can reduce body weight and serum cholesterol in HFD‐induced C57/BL6J mice (Li, Liu, et al., 2022; Liu et al., 2021). ApoE deficiency in humans is related to a reduced breakdown of atherogenic lipoproteins, promoting hypercholesterolemia and AS development (Ghiselli et al., 1981). In this study, 16 weeks later, HFD‐induced weight gain and dyslipidemia were observed in ApoE^−/−^ mice. At the same time, ACN slowed weight gain and reduced lipid accumulation and serum TC and LDL‐C levels. These results confirmed the hypolipidemic effect of ACN and indicated that ACN is essential in preventing CVD.

A high‐fat diet can alter the gut microbiome and lead to metabolic imbalances, including lipid and BA metabolism, caused by dysbiosis of gut microbiota (Wang et al., 2011). The gut‐liver axis plays an important role in maintaining metabolic homeostasis, especially since the liver is an important organ for regulating the balance of lipid metabolism (Ponziani et al., 2015). In the present study, a high‐fat diet accelerated lipid accumulation in the liver of ApoE^−/−^ mice, leading to hepatic steatosis, was significantly improved by the ACN treatment. Liu et al. (2021) demonstrated that the main monomer of ACN can exert anti‐obesity effects by inhibiting hepatic lipid synthesis, promoting lipolysis, and regulating the intestinal flora. A Previous study suggested that long‐term consumption of ACN has positive effects on the health of C57BL/6J mice and significantly regulates intestinal flora (Peng et al., 2020). Our previous study also suggested that ACN supplementation altered the gut microbiota composition in HFD‐fed C57BL/6J mice (Li, Liu, et al., 2022). In this study, the PCoA result revealed that the effects of ACN on intestinal structure of microbiota induced by HFD in ApoE^−/−^ mice were significant. ACN supplementation significantly enhanced the relative abundances of beneficial microbes like *Faecalibaculum_rodentium* and *Bifidobacterium_pseudolongum*. The indigenous murine gut microbiota, such as *Faecalibaculum_rodentium*, has been demonstrated to produce short‐chain fatty acids (SCFAs), which are known to inhibit intestinal tumorigenesis (Zagato et al., 2020). In the gut, SCFAs are among the most prominent microbial metabolites recognized for their pivotal role in modulating the host metabolism (Lin et al., 2012). *Bifidobacterium_pseudolongum* is a probiotic; previous research indicated that supplementation or enhancement of *Bifidobacterium_pseudolongum* protected against obesity induced by HFD (Bo et al., 2020). Moreover, a previous study found that *Bifidobacterium_pseudolongum* intervention could simultaneously increase the abundance of both *Bifidobacterium* and *Faecalibaculum* (Guo et al., 2022). We also found that ACN administration reversed the upregulated relative abundance of *Lachnospiraceae_bacterium_28–4*. Previous studies have also evinced that *Lachnospiraceae_bacterium_28–4* is which is effective in obesity‐linked microbiota composition (Zhu et al., 2017).

Several researches have demonstrated that BA metabolism and the maintenance of BA homeostasis have crucial implications for the control and cure of lipid disorders and the reduction of the risk of CVD (Pushpass et al., 2022). The gut microbiota controls the BA pool size and components ratio (Jia et al., 2018). BAs regulate intestinal flora, which prevents bacterial translocation and enhances mucosal barrier defense (Zhu et al., 2020). Studies in recent years have shown that crosstalk between BAs and the gut microbiota strongly influences host metabolism (Li, Wang, et al., 2022). BSH plays a crucial role in catalyzing the gateway reaction, the cleavage of the amino group from the conjugated BAs to generate unconjugated BAs (Batta et al., 1990; Jones et al., 2008). Interestingly, the oral administration of BSH inhibitors has demonstrated promise in the management of metabolic disorders, which achieves this by modulating intestinal FXR signaling through microbial function alteration and BA depolymerization reduction (Huang et al., 2019; Xie et al., 2020). Previous research has indicated that the theabrownin found in Pu‐erh tea exerts its effect on attenuating hypercholesterolemia by inhibiting the growth of BSH‐producing microbiota and increasing the level of conjugated BAs in the intestine (Huang et al., 2019). C57/BL6 mice gavaged with a single dose of a covalent BSH inhibitor displayed decreased BSH activity and unconjugated BAs levels, also increased conjugated BAs levels in feces (Adhikari et al., 2020). In our study, ACN treatment significantly affected the fecal BA profile induced by the HFD in ApoE^−/−^ mice. As expected, ACN supplementation notably increased in conjugated to unconjugated BAs ratio compared to the HFD group. The specific manifestations were an increase in conjugated BAs, including TCA and TαMCA, and a decrease in unconjugated BAs such as βMCA and 12keto_LCA. These results suggested that ACN might inhibit the BSH activity in regulating the compositional fecal BA pool in HFD‐induced ApoE^−/−^ mice.

Our findings show the gut microbiota and fecal BAs correlation to study their potential dependencies. The current study found a solid correlation between gut microbiota and fecal BA profile. Specifically, *Allobaculum*, *Bifidobacterium* enriched in the HFD + ACN group positively correlated with conjugated BAs, such as TCA and THDCA. Furthermore, the relative abundance of *Faecalibaculum* was positively associated with TCA and THDCA. TCA is known for glucose‐reducing effects on humans (Wu et al., 2013) and immunoregulatory properties in normal Kunming mice (Wang et al., 2013). THDCA is a natural 6α‐hydroxylated BA. Studies showed that THDCA alleviated ulcerative colitis and reduced the inflammatory response (Lv et al., 2023). ACN treatment resulted in a strong correlation between these BAs and bacteria with beneficial effects, suggesting that these BAs are associated with lipid‐lowering effects. However, in this study, some bacteria with BSH function, such as *Allobaculum* and *Bifidobacterium*, were enriched in the HFD + ACN group, which may indicate that we should conduct further research on the BSH activity of ileal intestinal microbiome and intestinal BA profile.

## 5 CONCLUSIONS

In conclusion, ACN exhibited a potential protective effect on hypercholesterolemia in ApoE^−/−^ mice induced by HFD, which may be related to the regulation of gut microbiota and fecal bile acid profile. Our study only highlighted the changes in gut microbiota and fecal BA profile after ACN intervention and has not yet involved the mechanisms of lipid and BA metabolism. Therefore, we will conduct further exploration and research. In addition, changes in BA pools at different sites and the associated metabolic pathways should also be investigated for a complete understanding of the effects of ACN on BA and lipid metabolism.

## AUTHOR CONTRIBUTIONS

**Meng Zhang:** Formal analysis (equal); investigation (equal); methodology (equal); writing – original draft (equal); writing – review and editing (equal). **Hui Li:** Formal analysis (equal); investigation (equal). **Tingting Tan:** Formal analysis (equal); investigation (equal); methodology (equal). **Lu Lu:** Methodology (equal); resources (equal). **Jia Mi:** Methodology (equal); resources (equal). **Abdul Rehman:** Writing – review and editing (equal). **Yamei Yan:** Funding acquisition (equal); methodology (equal); project administration (equal); writing – review and editing (equal). **Linwu Ran:** Conceptualization (equal); funding acquisition (equal); project administration (equal); supervision (equal); writing – original draft (equal); writing – review and editing (equal).

## FUNDING INFORMATION

This work was supported by the grants from the National Natural Science Foundation of China (NSFC, No. 81960588), Natural Science Foundation of Ningxia (2023A1434), and Ningxia Key Research and Development Program (2020BBF02006, 2021BEF02008).

## CONFLICT OF INTEREST STATEMENT

The authors declare no conflict of interest.

## ETHICS STATEMENT

Animal experiments were approved by the Ningxia Medical University Animal Ethics and Welfare Committee (IACUC‐NYLAC‐2023‐085).

## Data Availability

Data available on request from the authors.
